# Remediating Intractable Headache: An Effective Nonpharmacological Approach Employing Infralow Frequency Neuromodulation

**DOI:** 10.3389/fnhum.2022.894856

**Published:** 2022-07-08

**Authors:** Stella B. Legarda, P. Andreas Michas-Martin, Dana McDermott

**Affiliations:** Neurology, Montage Medical Group, Montage Health, Monterey, CA, United States

**Keywords:** intractable migraine, intractable tension headache, migraine theory, hypothalamic-limbic system dysfunction, infralow frequency modulation, chronic daily headache

## Introduction

The World Health Organization (WHO) has estimated that almost half the adult world population has experienced at least one headache within the past year. Headache is underestimated, under-recognized and under-treated, and only a minority of headache disorders are appropriately diagnosed by a health care provider (Pietrasik, 2016). Western medical therapies themselves may inadvertently induce headaches and, relatedly, medication-overuse headaches may ensue. The 3^rd^ edition of the international classification of primary headache disorders (ICHD-3) (Society, 2021) provides diagnostic criteria for migraine, tension-type headache (TTH), trigeminal autonomic cephalgias (TACs) (Table 1) and “other primary headache disorders” (Table 1).

**Table 1:** International classification of headache disorders, 3^rd^ edition (Society, 2021).

**A**
**3. Trigeminal autonomic cephalgias (TACs)**
**3.1 cluster headache** 3.1.1 Episodic cluster headache 3.1.2 Chronic cluster headache **3.2 Paroxysmal hemicrania** 3.2.1 Episodic paroxysmal hemicrania 3.2.2 Chronic paroxysmal hemicrania **3.3 Short-lasting unilateral neuralgiform headache attacks** 3.3.1 short lasting unilateral neuralgiform headache attacks with conjunctival injection and tearing (SUNCT) 3.3.1.1 episodic SUNCT 3.3.1.2 chronic SUNCT 3.3.2 short lasting unilateral neuralgiform headache attacks with cranial autonomic symptoms (SUNA) 3.3.2.1 episodic SUNA 3.3.2.2 chronic SUNA **3.4 hemicrania continua** 3.4.1 hemicrania continua, remitting subtype 3.4.2 hemicrania continua, unremitting subtype **3.5 probable trigeminal autonomic cephalgia** 3.5.1 probable cluster headache 3.5.2 probable paroxysmal hemicrania 3.5.3 probable short-lasting unilateral neuralgiform headache attacks 3.5.4 probable hemicrania continua
**B**
1. Headaches associated with physical exertion, including 4.1 *Primary cough headache*, 4.2 *Primary exercise headache*, 4.3 *Primary headache associated with sexual activity* and 4.4 *primary thunderclap headache*. 2. Headaches attributed to direct physical stimuli (consider to be primary headache disorders because they are brought on by physiological [non-damaging] stimuli), including 4.5 *Cold-stimulus headache* and 4.6 *External-pressure headache*. 3. Epicranial headaches (*ie*, head pain over the scalp), including 4.7 *Primary stabbing headache* and 4.8 *Nummular headache* as well as A4.11 *Epicrania fugax*. 4. Other miscellaneous primary headache disorders including 4.9 *Hypnic headache* and 4.10 *New daily persistent headache*.

The prevalence of migraine in the United States (U.S.) is high, suffered by 18% of women and 6% of men (Stewart et al., 1992). Refractory migraine headaches are characterized by greater headache pain frequency and a more significant array of comorbidities. The burden of migraine on society has been underestimated. Related disability and economic costs have recently been determined to fall on the patients and their workplace in the form of bedridden days and lost productivity; third-party payers assume less than 10% of migraine-related economic costs (Hu et al., 1999). In a recent investigation on the burden of non-communicable neurological disorders in the United States from 1990-2017, migraine was ranked third after stroke and Alzheimer disease (Feigin et al., 2021). In terms of prevalence, tension type headaches and migraine ranked first, with a rate change increase of 31.7 and 28.5% respectively in this time frame. Headache disorders are most common between ages 50–60 years, in the range of our most productive years (Stovner et al., 2014). The burden of headache-related disability is greatest for individuals with migraine (70%) compared to TTH (30%) (Stovner et al., 2007).

The pathogenesis of chronic migraine involves the maladaptation of “top-down” pain modulation and subsequent sensitization of the trigeminal system (Su and Yu, 2018). Migraine theory continues to evolve; participation of the trigeminal complex (cranial nerve V) remains relevant. We briefly discuss current concepts on migraine pathogenesis and the brain regions primarily involved: trigeminal complex, hypothalamus, and limbic system.

Traditional neurofeedback (NF) utilizing quantitative electroencephalogram (qEEG)-guided brain training protocols was demonstrated to be “dramatically effective” in managing recurrent migraine headache (Walker, 2011). Significant reductions in the frequency, severity and duration of headaches were also achieved in a group of healthcare professionals after suppressing theta (4–8 Hz), strengthening the sensorimotor rhythm (SMR, 12–15 Hz), and suppressing high beta (21–30 Hz) at T3 and T4 sites (Farahani et al., 2014). Another study utilizing traditional NF achieved 50% reduction of headaches in 70% of migraine patients, a superior outcome when compared to medical therapies (Stokes and Lappin, 2010). We share our 10-years' experience in remediating refractory primary headache disorders using infralow frequency brain training (ILF), a second-generation form of neurofeedback that is not qEEG-based and wherein scale-based training by way of conscious feedback is not utilized. An earlier proposed mechanism of ILF neurotherapy compellingly aligns with current theory in migraine pathogenesis; the work of earlier investigators on the slow cortical potential (SCP), modern migraine theory and their physiologic-neuroanatomic correlation is discussed.

## Background

Computer analyses of headache diagnosis in 600 patients referred to a neurology clinic aimed to (a) assess objectivity of the clinical diagnoses and (b) whether clinical diagnoses adequately represented the usual grouping of primary headache symptoms into five categories:

1) Classical migraine
2) Common migraine
3) Tension-vascular or “mixed” headache
4) Tension headache
5) Cluster headache

Overlap between common migraine, tension-vascular and tension headache symptoms were not adequately separated by clinical definitions; the authors concluded that *only cluster headache* was a true discrete entity, and that the other clinically defined categories represent different points in a continuum rather than being discrete entities. They concluded: “whether a single common mechanism underlies this headache spectrum, or whether two or more mechanisms interact to produce head pain can be determined only by further studies of the pathophysiology of headache” (Drummond and Lance, 1984). Thus, for most of these patients the cause of headache was unknown and their headache symptoms, except for cluster, were not categorically specific.

Chronic daily headaches (CDH), a diagnosis not listed officially in the ICHD, are defined as 15 or more episodes of headache in a month occurring for at least three months (Murinova and Krashin, 2015). Most CDH transform from episodic headache disorders. Chronic migraine headache disorders share features such as sensitization of the trigeminal system, structural and functional alterations in the brain and environmental factors, among them medication overuse (Su and Yu, 2018). Patients with CDH are like patients with chronic migraine who report significant comorbidities such as anxiety, depression, sleep disorders, digestive concerns, addictions, and other complaints.

## Neuroanatomy of Migraine and Cluster Headaches

Headache mechanisms have long implicated the trigeminal nuclear complex (Liveing, 1873; Gowers, 1888); this “trigeminal theory” best matches the unilateral presentation of most migraineurs and cluster headache sufferers. Recent migraine theory continues to implicate trigeminal pathways and continues to evolve. At one time the rostral dorsal pons (positioned near cranial nerve V) was generally agreed to be the generator of pain during migraine without aura based on functional imaging studies (Weiller et al., 1995). In cluster headache the posterior hypothalamus is implicated (May et al., 1998) and deep brain stimulation (DBS) of the posterior hypothalamus has resulted in relief from chronic cluster headache (Franzini et al., 2003). Extensive anatomic investigations of the trigeminal nociceptive pathways have been (Capra and Dessem, 1992) and continue to be elucidated (Kagan et al., 2013). Advanced neuroimaging techniques provide clearer anatomic evidence that the clinical symptoms and cyclical nature of both primary headache disorders reflect a dysfunctional *hypothalamic-limbic circuitry* (May and Burstein, 2019) (Table 2).

**Table 2:** Symptoms in primary headache syndromes are referrable to the hypothalamic-limbic circuitry (Gowers, 1888).

**Hypothalamus**	**Headaches Vision changes**	**Sleep disorders**	**Constant thirst**	**Nausea, vomiting**	**Lack of sex drive**	**Fatigue** **Anhedonia**	**Temperature instability**	**Obesity**
Limbic system	Amotivational	Aggression, Anger	Anxiety	Stress	Memory changes	Attention deficit disorders	Mood swings	Addictions

### Hypothalamus (Function: Homeostasis and Regulation)

This structure surrounds the 3^rd^ ventricle immediately below the thalamus (hence its name) limited anteriorly by the optic chiasm and anterior commissure and posteriorly by the mamillary bodies. Together the hypothalamus and the limbic system maintain homeostasis by exerting control on the endocrine and autonomic nervous systems, including our emotions, motivations, and behavior. Clinically relevant effects include body temperature, blood pressure, electrolyte balance, energy metabolism, reproduction, and the stress response. Hypothalamic function is also involved in addiction, anxiety disorders, and weight maintenance. Some investigators assume the hypothalamus regulates the limbic system (Stankewitz et al., 2021). Major visceral afferent connections to the hypothalamus arrive from the nucleus tractus solitarius, which also receives a convergence of inputs from cranial nerves VII, IX and X and relays information to the limbic system. A notable connection is the *trigeminohypothalamic tract* originating from neurons in the trigeminal nucleus caudalis (Malick and Burstein, 1998) which receives afferents from head and neck structures including the meninges and descends to the third cervical level in the spinal cord receiving nociceptive cervical afferents. There are three main mechanisms in the hypothalamus that make its function analogous to servo-control systems: it receives sensory information, it compares sensory inputs with biological set points, and it adjusts the array of autonomic, endocrine, and behavioral responses with the purpose of maintaining homeostasis (Kandel et al., 2000).

### Limbic System (Function: Processing of Fear, Stress Reactivity, Learning and Memory)

Put simply, the limbic system comprises anatomic regions that link the cortex with subcortical structures, thus it is comprised of cortical areas, subcortical areas, and diencephalic structures. Its identity as a distinct entity is controversial; it is only one of many brain regions that regulate visceral autonomic processes. The limbic system is said to mediate between autonomic reactions and the cognitive evaluation of aversive sensory inputs (Wager et al., 2008). Dopaminergic projections from the limbic system modulate the nucleus accumbens (basal region of forebrain and part of basal ganglia) which plays a role in sexual arousal and the “high” from use of recreational substances, believed to be a basis for much of addiction psychopathology. Limbic circuitry is involved in motivation, emotion, learning and memory; its close relationship with hypothalamic function in homeostasis allows us to transcend feelings, judgment- perceptions and emotions to offset potential imbalances.

### Top-Down Regulation of Pain

The brainstem periaqueductal gray matter (PAG) is believed to be involved in the top-down, descending modulation of the trigeminal nuclei. The PAG has strong connections with the hypothalamus and limbic forebrain structures including the amygdala and projects to the rostral ventral medulla where pain transmission is inhibited or facilitated via direct projections to the spinal and medullary dorsal horn laminae critical in nociceptive function (Heinricher et al., 2009).

## Modern Migraine Theory

Modern theory development targets the hypothalamus as the central area affected in both migraine and cluster headaches since it is the hypothalamus, serving in its role to maintain homeostasis, that determines which relay pathway will dominate the firing of a trigeminovascular thalamic neuron at any given time (May and Burstein, 2019). The networks through which the hypothalamus serves this role in migraine are both influenced by, as well as sub-serve, multiple functions that include setting brainstem oscillatory functions, sensory thresholds, and pain modulation at all levels of the neuroaxis. In longitudinal data obtained by functional magnetic resonance imaging (fMRI) from 12 migraineurs, perfusion changes over the migraine cycle occurred mainly in limbic structures with lower hypothalamic connectivity noted during the headache phase, leading these investigators to propose that an increasing loss of hypothalamic control over the limbic structures increases the susceptibility of limbic neurons to migraine triggers (Stankewitz et al., 2021).

## Theory of Infralow Frequency Neuromodulation

Infra-slow brain oscillations (<0.5 Hz) were first reported by Russian scientist, Aladjalova (1956). Aladjalova characterized it further in 1964 by demonstrating how stimulation of the reticular activating system (RAS) immediately elicits arousal in the cortical EEG (“rapid regulatory system”) but has no effect on this infraslow activity (Aladjalova, 1964). Aladjalova additionally demonstrated that stimulation of the ventromedial part of the hypothalamus intensified infraslow cortical activity within 30–40 min (“slow regulatory system”). She conjectured: “this phenomenon reflects the activity of the *slow control system* of the brain… not only to automatically adjust the system in keeping the internal environment constant but actively to establish a new level of activity.” The slow control system, with which we engage during infralow frequency-based neurofeedback, is therefore dynamically associated with the hypothalamus, either regulated by it *or vice versa*.

Hughes et al. (2011) more recently demonstrated the electrophysiological characteristics of infraslow (<0.02 Hz) oscillations recorded from corticothalamic slices of cats. They determined the origin of this phenomenon to be *extra-neuronal* and dependent on the ATP from astrocytes (Hughes et al., 2011). We previously proposed that ILF brain training engages with resting state networks in the brain (Legarda et al., 2011) having similar frequency characteristics (Damoiseaux et al., 2006). The physiologic importance of astrocytes in circadian slow frequency biologic regulation has been reported (Womac et al., 2009). More recently investigators have begun to stress the critical role of astrocytes in neuromodulation, synaptic plasticity, and learning (De Pittà et al., 2016).

As stated, there is evidence that migraine and cluster headache disorders reflect a dysregulation or impaired modulation of hypothalamic networks (Gowers, 1888; May and Burstein, 2019). By its direct engagement with and/or promotion of the slow cortical potential, consistent *infralow frequency* brain training “exercise” re-regulates and restores healthy modulation of the hypothalamic-limbic circuitry (and of the slow regulatory system).

## Functional MRI Studies

**Figure 1:**
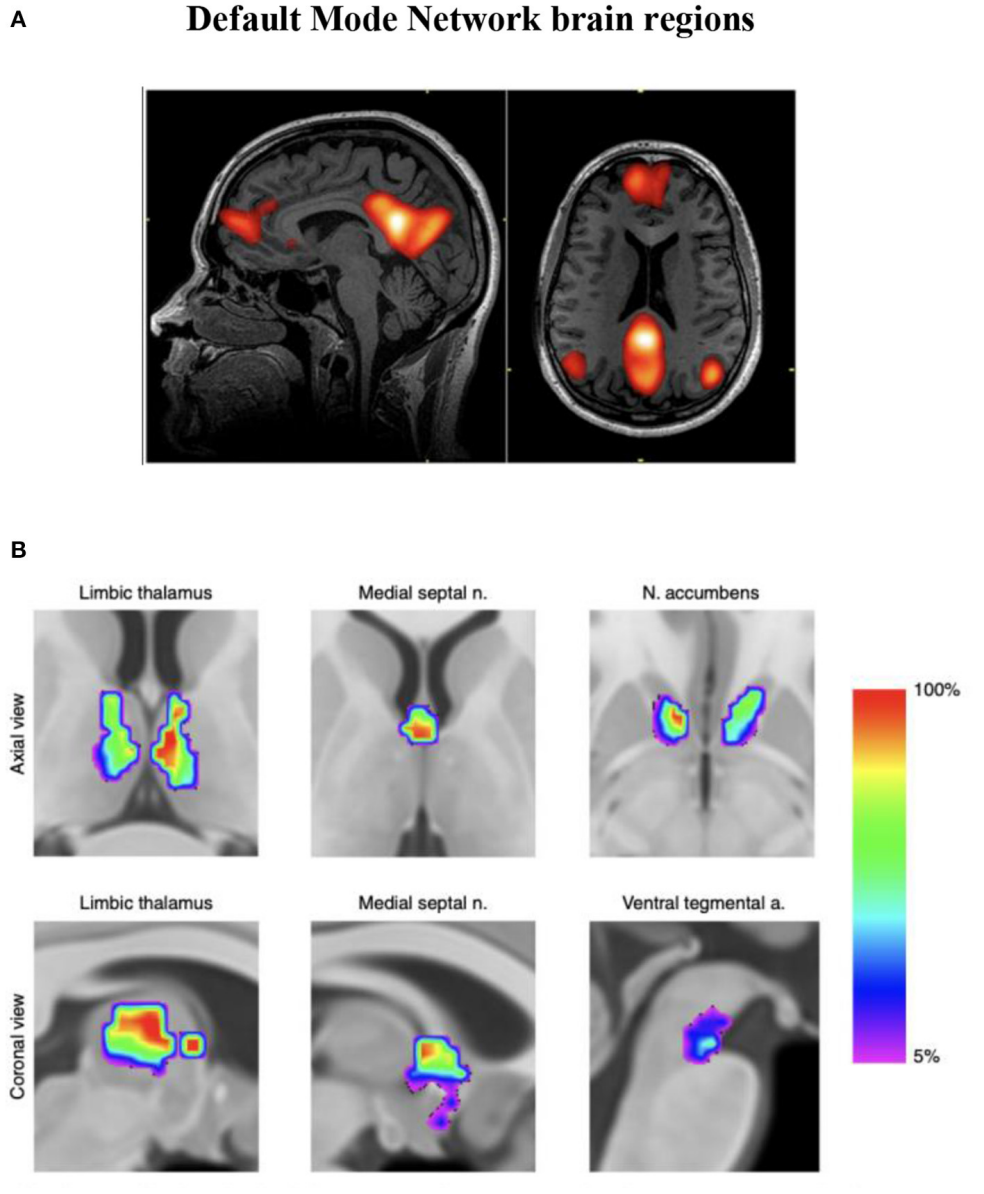
**(A)** Functional MRI demonstration of the default mode network (DMN). **(B)** Discrete neuroanatomical model of the DMN showing coactivation of brainstem regions. This map is freely available at https://www.nature.com/articles/s42003-019-0611-3/figures/4. n = 20 participants. Improved neuroanatomical model of the default-mode network shows co-activation of brainstem and midbrain regions with DMN hubs. This reconciles previous neuroimaging and neuropathological findings (Alves et al., 2019).

Infra-slow brain oscillations are also observed as fluctuations in the BOLD (blood-oxygen-level-dependent) signal consistently measured by fMRI during awake, resting states in healthy humans (Damoiseaux et al., 2006). The first of these “resting state networks” (RSNs) reliably observed in brain regions during task-negative alert states was characterized as the default mode network (DMN) (Buckner et al., 2008) (see Figure 1A), which has been further described to oscillate at a frequency < 0.1 Hz and to modulate other functional networks (Fox and Raichle, 2007). Resting state functional MRI studies of migraine patients (in between attacks) have demonstrated mixed findings evidencing disruption of RSNs (Tessitore et al., 2013; Xue et al., 2013; Zhang et al., 2016). During a migraine attack, pain intensity has been associated with reduced connectivity between DMN and insula cortex (Coppola et al., 2017).

**Figure 2:**
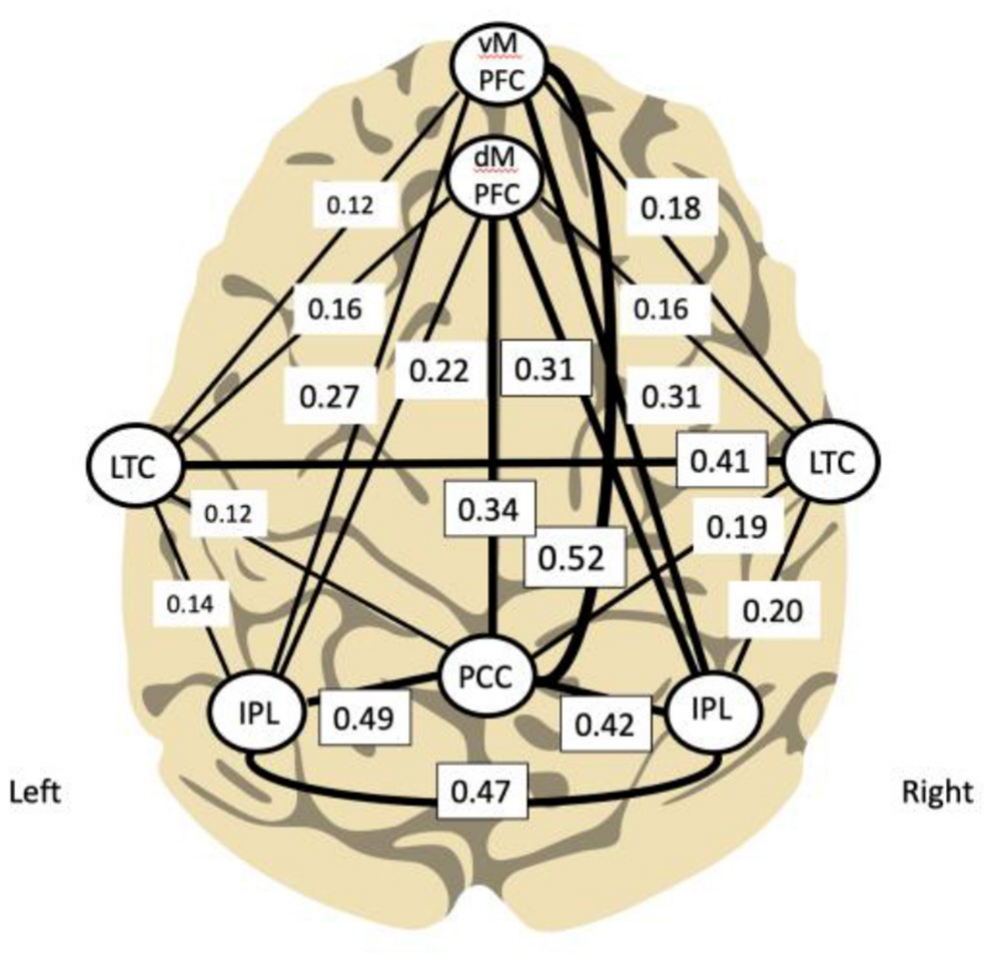
Principal functional hubs and connectivity within default mode network (Buckner et al., 2008; Other and Othmer, 2019) (modified). Density of connectivity is correlated with line thickness.

Using functional connectivity analysis, Buckner and colleagues have delineated the hubs and subsystems of the DMN in the brain demonstrating its relative denser connectivity within the right hemisphere (see Figure 2). Interestingly, training the right side first was determined relevant during development of the ILF method; this practice approach remains the standard.

Also relevant to our discussion of RSNs is the salience network (SN) believed to process emotions, pain, and interoceptive-autonomic states (Otti et al., 2013). With key nodes in both insular cortices the SN detects behaviorally “salient” inputs through the coordination of neural resources that involve communication with visceromotor central pattern generators from subcortical structures including the hypothalamus and periaqueductal gray matter (Uddin, 2015). Decreased hypothalamus-SN coactivations have been found in patients with cluster headache, believed to reflect a defective central pathway of pain control and autonomic nervous system dysregulation (Qiu et al., 2015).

Unlike in the cortical and subcortical regions, *brainstem* fMRI networks have only recently been identified (Cauzzo et al., 2022). Using an approach different to most other investigators of resting state functional connectivity studies, abnormal amplitude increases in low frequency oscillations (or LFOs, 0.198–0.073 Hz) were demonstrated in thalamocortical networks of migraine patients in the interictal (between acute headaches) phase compared to controls. In their identification of thalamic LFOs (in both migraine and controls) these investigators hypothesized *a priori* that projections from the thalamus would be reflected in a core of low frequency cortical activity. They demonstrated projected thalamocortical relay regions to show disruptions in LFO; they also showed that the only subcortical regions showing altered IFO activity were the hypothalamus and thalamus. In migraine patients the intrinsic LFO activity favored the lower frequency state and was associated with headache attack rate, drawing their conclusion that there exists an abnormal interictal state of thalamocortical dysrhythmia in individuals with migraine (Hodkinson et al., 2016). More recently a longitudinal study across the migraine cycle of brainstem functional oscillations demonstrated marked increase brainstem variability during the 24-h period preceding a migraine attack (Meylakh et al., 2021). Brainstem areas demonstrating the greatest increase were the spinal trigeminal nucleus and dorsal pons and were characterized by increased power between 0.03–0.06 Hz.

The reproducibility of RSNs between subjects by fMRI investigators is what suggests an anatomical basis to the brain connectome (Biswal et al., 1995). We mention the RSNs here given their oscillatory frequency characterization; we suspect the ILF neuromodulation technique engages them less selectively given that large networks (such as these imaged by fMRI) are recruited during slow oscillations (Steriade, 2001; Steriade and Timofeev, 2003) and by the slow control system (Aladjalova, 1964).

Endeavors aimed at visualizing intrinsic neural networks continue to evolve. An improved association with ILF neuromodulation (whereby restoring or recovering previously normal innate regulation is our heuristic approach) is anticipated as fMRI investigations of subcortical and brainstem regions improve (see Figure 1B). The fMRI technique remains dependent on blood oxygen levels (hemoglobin state differences in magnetic susceptibility; BOLD phenomenon) reflecting vessel/capillary metabolism; it is not an electrophysiologic signature. Developments in fMRI technique to reflect *blood-brain barrier* metabolism (if doable) would disclose astrocytic networks potentially allowing us to visualize metabotropic pathways reflecting gliotransmission, recordable today as infraslow oscillations (ISO) (Hughes et al., 2011).

## ILF Brain Training to Remediate Primary Headache Disorders

In our neurology practice ILF brain training has provided relief to primary headache sufferers refractory to standard medical management (regardless of type), including those who have failed advanced pharmacological interventions (botulinum toxin and anti-CGRP injections). We speculate that refractory primary headache syndromes reflect a dysregulated state or chronic instability of hypothalamic-trigeminal connections (top-down dysmodulation) (Su and Yu, 2018), and that ILF brain training effects the re-regulation of hypothalamic (and limbic) networks.

### Patient Profiles

Headaches may present a “tip of the iceberg” phenomenon in many patients who often disclose significant comorbidities. Along with the headache complaint neurocognitive and even physiologic complaints may present, while illness chronicity is dominated by the migraine history. To illustrate our approach in managing these disorders with ILF brain training, here are three clinical groups each with a theoretical example of an intractable headache presentation:

**Group A**: “overwhelmed limbic system” - 36 year old business executive with chronic depression and PTSD on multiple medications, is referred by her counselor for debilitating headaches being managed by her neurologist with high dose topiramate (100 mg BID) without relief. After 20 sessions of neurofeedback, she is headache free, and over time becomes medication free for headache control (while continuing psychoactive medications).

**Group B**: “overwhelmed hypothalamic system” - 42 year old nurse has frequent work absences for the past 5 years because of severe migraine attacks; these persist on a recurring basis despite anti-CGRP management, amitriptyline prophylaxis and zonisamide prophylaxis in addition to prescriptive abortive agents (triptans, analgesics). He has failed topiramate prophylaxis and botulinum toxin injections and obtains only moderate relief from intermittent occipital nerve blocks. After just 5 sessions of ILF brain training his headache frequency begins to remit to mild episodes rarely; he has not used prescribed abortive measures for weeks. Problems remain at work; after passing a basic cognitive assessment he is determined to have attention deficit disorder. Low-dose methylphenidate is initiated and over time, as he completes his prescribed ILF brain training sessions all other medications can be subsequently and gradually weaned. He is maintained on OTC abortive medications alone as needed for rare headaches.

**Case C**: “disrupted hypothalamic-limbic circuitry” - 19 year old high school graduate (who had played football) sustained closed head trauma and significant postconcussion symptoms after an accident with his skateboard. Three months later he still complained of (worsening) headaches, brain fog, and forgetfulness. Six months later, unable to gain relief from common pharmacologic agents (acetaminophen, ibuprofen, amitriptyline and topiramate) he is referred for ILF neuromodulation and fully recovers his previous wellbeing after 15 sessions of prescribed ILF brain training. He is weaned from and successfully discontinues all prescribed medications.

Our10-year experience managing intractable headache disorders with ILF has allowed for less reliance not only on polypharmacy, thereby preventing the confounds of medication overuse but also on specific headache diagnostic categories. It is the symptom profile (Table 2) that serves best to identify who will benefit from re-regulation with ILF. Patients with medically refractory headache disorders tend not to fall neatly into some headache type. We make no attempt here to describe a distinct headache cohort; each refractory headache patient presents uniquely from the next. In the realm of our ILF practice intractable headache is more a symptom than a diagnosis; most patients present along the spectrum of the three anatomically designated headache groups described above. Not infrequently, other primary maxillofacial and cervicogenic musculoskeletal disorders are the cause. This is our approach to understanding and managing the dysregulated brain, therefore, almost irrespective of symptom presentation. *Objective* symptom tracking by our doctors over subsequent clinic visits (not subjective recollection as is typical of standard patient questionnaires) is important (Legarda et al., 2022). Given every human experience (and brain) is unique the ILF neurotherapy approach is necessarily further tailored to each individual.

In brief, chronic headache disorders are to some greater or lesser degree clinical representations of cerebral dysregulation, have a fairly consistent repertoire in presentation (Table 2) and our task is to re-regulate the CNS to achieve stability and restore homeostasis. A heuristic approach relating current neuroscience understanding with *patient-based* evidence is important. Integrative therapy involves typical migraine management approaches in tandem with ILF and relevant referrals for physical therapy and even surgery. In our experience at times dramatic improvement and even resolution of headache disorders in tandem with the reduced need for polypharmacy are frequently achieved.

## ILF Brain Training Protocol for Medically Refractory Headache Disorders

Headache disorders are by their nature an intermittent, episodic disruption in the life of patients and are considered “instabilities”, and T4-T3 is the preferred initial and inter-hemispheric training site (Farahani et al., 2014). Accessing the T4-T3 placement for neuromodulation promotes balanced training and global, bi-hemispheric stability. State regulation being a second key burden (including central arousal, mood, vegetative function), T4-P4 is a necessary second site to train [This right temporal-parietal primary training site was arrived at in a trial-and-error, patient-based approach by the developers of the ILF method over years (Othmer, 2019)]. The training strategy is to lower the training frequencies over consecutive visits; the lower the frequency the stronger the training. Many patients report an optimal response frequency at which they train for the remainder of their treatment. The total number of sessions, each for 50 min, varies in requirement, ranging from 20–40; some require, or request, more.

When most symptomatic instabilities and physiologic dysregulations are remediated, we add slow synchrony training accessing a Pz + Fz placement to engage with the anteroposterior hubs of the DMN (see Figure 1A) more directly at the end of later sessions. This training site appears to improve chronic sleep difficulties. For those more chronically affected often with autonomic instabilities we additionally train the insula region at T4-F8.

The typical headache training protocol montage sequence looks like this:

T4-T3 (bipolar training)
T4-P4 (bipolar training)
PZ+FZ (synchrony training)

We employ additional case specific training sites; thus, for Case B we would address ADD/ADHD and train additionally at T4-Fp1 and T3-Fp1. In cases with observable autonomic instabilities we train additionally at T4-F8.

Each neurofeedback session lasts 50 min allowing us to train 10 min at each site.

For Case C we initiate ILF training with the concussion protocol:

T4-O2, T4F-P2, T4-T3, T3-O1, T3-FP1.

### ILF Brain Training for Post-concussion Headache

Case C represents the more youthful form of post-concussion/ posttraumatic headache disorders. In addition to reporting severe headaches, patients of all ages variably present with cognitive changes, mood changes, ADD/ADHD, memory difficulties, brain fog, sleep disorders, non-specific visual disturbances, often severe anxiety, anhedonia and in older patients even features of parkinsonism. This rather catastrophic form of headache disorder seems to resolve more readily using our more global “concussion protocol” in ILF brain training (Legarda et al., 2022).

In our experience patients with postconcussion symptoms (PCS) (Gasquoine, 1997) and persistent (greater than 3 months) post-concussion symptoms (PPCS) (Bigler, 2008) do not initially tolerate parietal training. Diffuse axonal injury (DAI) impairments following significant concussion have been reported to disrupt normal DMN function (Churchill et al., 2018). Based on our patient experience, these discrete axonal connections affected by DAI are more likely to be optimally relieved, restored, and more readily recovered (paralleling more rapid improvements in patient symptoms) by long distance intra*-*hemispheric training sites (T4-O2, T4-Fp2, T3-O1and T3-Fp1). The T4-T3 site provides interhemispheric stability, as with other headache syndromes, and is well tolerated by post-concussion patients. Not infrequently we refer our patients with PPCS for neuropsychological evaluation. After physiological clinical improvements are reported, we find patients better able to tolerate training at more discrete cognitive domain sites to address their reported and formally detected deficits.

After at least five initial sessions with the concussion protocol (we recommend more for severe cases and after beta suppression is achieved as indicated by the session trend graph) we proceed to shorter-distance training sites as the patient's symptoms and neurocognitive evaluations dictate, almost invariably starting at T4-P4 for general arousal regulation and parietal calming. As mentioned, the DMN is invariably impacted by concussion, and we sometimes conclude with slow synchrony (0.05 Hz or less) training at Pz + Fz; we find this especially benefits patients with sleep disturbances. Synchrony training is potentially destabilizing; we do this only after interhemispheric and global stability is achieved with bipolar ILF training, evidenced by suppression of the beta (<13 Hz) and hi-beta (>20 Hz) bands in EEG frequency trend graphs recorded during the shorter-distance training session (In our experience young children do not tolerate synchrony training, at least at the frequencies currently available in the Cygnet^*^ synchrony program software.).

## Summary and Closing Remarks

Headache and non-headache symptoms of migraine reflect disturbances in homeostasis under hypothalamic-limbic control. Current migraine theory targets hypothalamic dysregulation (Gowers, 1888; May and Burstein, 2019). The brain's slow cortical potential (SCP) has been demonstrated to reveal a direct hypothalamic association with the “slow control system” (Aladjalova, 1964). Brain training of these infra-low frequencies engages with the slow control system. Particularly when this training is performed at the individualized optimal repsonse frequency, and in a professionally-supervised setting, it allows individuals with dysregulated slow control systems (specifically hypothalamic-limbic metabotropic networks) to recover and restore improved homeostasis. It is a growing understanding that brain training, or neuromodulation involves metabotropic slow signaling pathways dependent upon and accomplished by mostly astrocytic networks; ILF brain training strengthens these pathways promoting neuroplasticity and learning.

Infralow frequency neuromodulation guides medical management toward additional therapeutic targets when underlying causes and/or triggers become evident. Physician-driven objective symptom tracking is imperative. While many patients benefit from ILF to alleviate their headache and other symptoms reflecting a dysregulated central nervous system, some require an extensive multifactorial management approach. Physical therapy interventions are frequently recommended for musculoskeletal based disorders impacting chronic afferent volleys to the trigeminal nucleus caudalis. Dental, maxillofacial, cervical, or cervical-occipital spine procedures may need to be considered requiring appropriate referral services. Supportive counseling and life coaching also form an important part of integrative therapy.

Our neurology practice considers ILF neurotherapy an essential tool in the integrative management of refractory migraine, trigeminal autonomic cephalgia and other primary headache disorders. Providing ILF neurotherapy as a non-pharmacological and non-surgical option early to our patients with chronic headache disorders reduces emergency room visits, prevents medication-overuse sequelae and builds resilience to potential anesthesia effects from any required surgical intervention. Our more than 10 years of experience with ILF neurotherapy in managing debilitating headache syndromes frames our projection that the broader implementation of this method, in conjunction with medical and adjunct therapies will lead to decreases in morbidity, functional impairment, bedridden days and lowered work productivity for chronic migraineurs, thereby reducing the socioeconomic burden of migraine.

## Author Contributions

SL: 75% manuscript preparation and 100% review of supportive literature. PM-M: 15% manuscript writing and editing and ILF neurotherapy collaboration. DM: 10% manuscript writing and editing and ILF neurotherapy collaboration. All authors contributed to the article and approved the submitted version.

## Conflict of Interest

The authors declare that the research was conducted in the absence of any commercial or financial relationships that could be construed as a potential conflict of interest.

## Publisher's Note

All claims expressed in this article are solely those of the authors and do not necessarily represent those of their affiliated organizations, or those of the publisher, the editors and the reviewers. Any product that may be evaluated in this article, or claim that may be made by its manufacturer, is not guaranteed or endorsed by the publisher.

## References

[B1] Aladjalova N. (1956). Infra-slow rhythmic changes of the brain electrical potential. Biophysica. 1, 127–136.

[B2] Aladjalova N. (1964). Slow electrical processes in the brain. Progr. Brain Res. 7, 156–206.

[B3] Alves P. N. Foulon C. Karolis V. Bzdok D. Margulies D. S. Volle E.. (2019). An improved neuroanatomical model of the default-mode network reconciles previous neuroimaging and neuropathological findings. Commun. Biol. 370, 1–14. 10.1038/s42003-019-0611-3 31633061 PMC6787009

[B4] Bigler E. (2008). Neuropsychology and clinical neuroscience of persistent post-concussive syndrome. J. Int. Neuropsychol. Soc. 14, 1–22. 10.1017/S135561770808017X 18078527

[B5] Biswal B. Yetkin F. Haughton V. Hyde J. (1995). Functional connectivity in the motor cortex of resting human brain using echo-planar MRI. Magn. Reson. Med. 34, 537–541. 10.1002/mrm.1910340409 8524021

[B6] Buckner R. Andrews-Hanna J. Schacter D. L. (2008). The brain's default network: anatomy, function and relevance to disease. Ann. New York Acad. Sci. 1124, 1–38. 10.1196/annals.1440.011 18400922

[B7] Capra N. Dessem D. (1992). Central connections of trigeminal primary afferent neurons: topographical and functional considerations. Crit. Rev. Oral Biol. Med. 4, 1–52. 10.1177/10454411920040010101 1457683

[B8] Cauzzo S. Singh K. Stauder M. García-Gomar M. G. Vanello N. Passino C.. (2022). Functional connectome of brainstem nuclei involved in autonomic, limbic, pain and sensory processing in living humans from 7 Tesla resting state fMRI. Neuroimage 250, 118925. 10.1016/j.neuroimage.2022.118925 35074504 PMC8885980

[B9] Churchill N. Hutchison M. Graham S. Schweizer T. (2018). Connectomic markers of symptom severity in sport-related concussion: Whole-brain analysis of resting-state fMRI. Neuroimage: Clin. 18, 518–526. 10.1016/j.nicl.2018.02.011 29560308 PMC5857899

[B10] Coppola G. Di Renzo A. Tinelli E. Di Lorenzo C. Scapeccia M. Parisi V.. (2017). Resting state connectivity between default mode network and insula encodes acute migraine headache. Cephalalgia 38, 846–854. 10.1177/0333102417715230 28605972

[B11] Damoiseaux J. S. Rombouts S. A. R. B. Barkhof F. Scheltens P. Stam C. J. Smith S. M.. (2006). Consistent resting-state networks across healthy subjects. PNAS 103, 13848–13853. 10.1073/pnas.0601417103 16945915 PMC1564249

[B12] De Pittà M. Brunel N. Volterra A. (2016). Astrocytes: Orchestrating synaptic plasticity? Neuroscience 323, 43–61. 10.1016/j.neuroscience.2015.04.001 25862587

[B13] Drummond P. Lance J. (1984). Clinical diagnosis and computer analysis of headache symptoms. J. Neurol. Neurosurg. Psychiat. 47, 128–133. 10.1136/jnnp.47.2.128 6707652 PMC1027680

[B14] Farahani D. Tavallaie S. Ahmadi K. Ashtiani A. (2014). Comparison of neurofeedback and transcutaneous electrical nerve stimualtion efficacy ont reatment of primary ehadaches: a randomized controlled clinical trial. Iran Red Crescent Med. J. 16, e17799. 10.5812/ircmj.17799 25389484 PMC4222010

[B15] Feigin V.L. Vos T. Alahdab F. Amit A.M.L. Bärnighausen T.W. Beghi E.. (2021). Burden of neurological disorders across the US from 1990-2017 a global burden of disease study. JAMA Neurol. 78, 165–176. 10.1001/jamaneurol.2020.4152 33136137 PMC7607495

[B16] Fox M. Raichle M. (2007). Spontaneous fluctuations in brain activity observed with functional magnetic resonance imaging. Nat. Rev. Neurosci. 8, 700–711. 10.1038/nrn2201 17704812

[B17] Franzini A. Ferroli P. Leone M. Broggi G. (2003). Stimulation of the posterior hypothalamus for treatment of chronic intractable cluster ehadaches: first reported series. Neurosurgery 52, 1095–1101. 10.1227/01.NEU.0000057698.29634.D6 12699552

[B18] Gasquoine P. (1997). Postconcusison Symptoms. Neuropsychol. Rev. 7, 77–85. 10.1023/B:NERV.0000005945.58251.c0 9253770

[B19] Gowers W. (1888). A Manual of Diseases of the Central Nervous System. Philadelphia: P. Blakiston, Son and Co. 10.2307/1411377

[B20] Heinricher M. Tavares I. Leith J. Lumb B. (2009). Descending control of nociception: specificity, recruitment and plasticity. Brain Res. Rev. 60, 214–225. 10.1016/j.brainresrev.2008.12.009 19146877 PMC2894733

[B21] Hodkinson D. J. Wilcox S. L. Veggeberg R. Noseda R. Burstein R. Borsook D.. (2016). Increased Amplitude of Thalamocortical Low-Frequency Oscillations in Patients with Migraine. J. Neurosci. 36, 8026–8036. 10.1523/JNEUROSCI.1038-16.2016 27466345 PMC4961783

[B22] Hu X. Markson L. Lipton R. Stewart W. Berger M. (1999). Burden of migraine in the Untied States. Arch. Internal Med. 159, 813–818. 10.1001/archinte.159.8.813 10219926

[B23] Hughes S. Lorincz M. Parri H. Crunelli V. (2011). Infra-slow (<0.1 Hz) oscillations in thalamic relay nuclei: basic mechanisms and significance to health and disease states. Prog. Brain Res. 193, 145–162. 10.1016/B978-0-444-53839-0.00010-7 21854961 PMC3173874

[B24] Kagan R. Kainz V. Burnstein R. (2013). Hypothalamic and basal ganglia projections to the posterior thalamus: Possible role in modulation of migraine headache and photophobia. Neuroscience 248, 359–368. 10.1016/j.neuroscience.2013.06.014 23806720 PMC3858508

[B25] Kandel E. R. Schwartz J. H. Jessell T. M. Siegelbaum S. Hudspeth A. J. Mack S. (2000). Principles of Neural Science. New York: McGraw-hill.

[B26] Legarda S. Lahti C. Michas-Martin A. McDermott D. (2022). Use of novel concussion protocol with infralow frequency neuromodulation in prolonged postconcussion syndrome. Front. Human Neurosci. 15:763580. 10.3389/fnhum.2022.894758 35685335 PMC9170890

[B27] Legarda S. McMahon D. Othmer S. Othmer S. (2011). Clinical neurofeedback: case studies, proposed mechanism, and implications for pediatric neurology practice. J. Child Neurol. 26, 1045–1051. 10.1177/0883073811405052 21576401

[B28] Liveing E. (1873). On Megrim, Sick-Headache, and Some Allied Disorders. A Contribution to the Pathology of Nerve-Storms. London: Arts and Boeve Nijmegen.

[B29] Malick A. Burstein R. (1998). Cells of origin of the trigeminohypothalamic tract in the rat. J. Comp. Neurol. 400, 124–144. 10.1002/(SICI)1096-9861(19981012)400:1<125::AID-CNE9>3.0.CO;2-B 9762871

[B30] May A. Bahra A. Buchel C. Frackowiak R. Goadsby P. (1998). Hypothalamic activation in cluster headache attacks. Lancet 351, 275–278. 10.1016/S0140-6736(98)02470-2 9690407

[B31] May A. Burstein R. (2019). Hypothalamic regulation of headache and migraine. Cephalgia 39, 1710–1719. 10.1177/0333102419867280 31466456 PMC7164212

[B32] Meylakh N. Marciszewski K. K. Di Pietro F. Macefield V. G. Macey P. M. Henderson L. A. (2021). Brainstem functional oscillations across the migraine cycle: A longitudinal investigation. Neuroimage: Clin. 30, 1–10. 10.1016/j.nicl.2021.102630 33770547 PMC8024773

[B33] Murinova N. Krashin D. (2015). Chronic daily headache. Phys. Med. Rehabil. Clin. N. Am. 26, 375–389. 10.1016/j.pmr.2015.01.001 25952071

[B34] Other S. Othmer S. (2019). Toward a theory of infralow frequency neurofeedback, in Restoring the Brain. Francis and Taylor. p. 65–70.

[B35] Othmer S. (2019). Protocol Guide for Neurofeedback Clinicians (7th Edition). Woodland Hills, CA: EEG Institute.

[B36] Otti A. Guendel H. Wohlschlager A. Zimmer C. Noll-Hussong M. (2013). Frequency shifts in the anterior default mode network and the salience network in chronic pain disorder. BioMed. Central Psychiat. 13, 1–9. 10.1186/1471-244X-13-84 23497482 PMC3616999

[B37] Pietrasik W. (2016). Headache disorders. (World Health Organization) Available online at: https://www.who.int/news-room/fact-sheets/detail/headache-disorders (accessed April 8, 2016).

[B38] Qiu E. Tian L. Wang Y. Ma L. Yu S. (2015). Abnormal coactivation of the hypothalamus and salience network in patients with cluster headache. Neurology 84, 1402–1408. 10.1212/WNL.0000000000001442 25746559

[B39] Society I. H. (2021). The International Classification of Headache Disorders 3rd edition. Available online at: https://ichd-3.org (accessed June 6, 2022).

[B40] Stankewitz A. Keidel L. Rehm M. Irving S. Kaczmarz S. Preibisch C.. (2021). Migraine attacks as a result of hypothalamic loss of control. Neuroimage: Clin. 32, 102784. 10.1016/j.nicl.2021.102784 34425551 PMC8379646

[B41] Steriade M. (2001). Impact of network activities on neuronal properties in corticothalamic systems. Am. Physiol. Soc. 86, 1–39. 10.1152/jn.2001.86.1.1 11431485

[B42] Steriade M. Timofeev I. (2003). Neuronal plasticity in thalamocortical networks during sleep and waking oscillations. Neuron 37, 563–576. 10.1016/S0896-6273(03)00065-5 12597855

[B43] Stewart W. Lipton R. Celentano D. Reed M. (1992). Prevalence of migraine ehadaches in the United States: relation to age, income, race and other sociodemographic factors. JAMA 267, 64–69. 10.1001/jama.1992.03480010072027 1727198

[B44] Stokes D. A. Lappin M. S. (2010). Neurofeedback and biofeedback with 37 migraineurs: a clinical outcome study. Behav. Brain Funct. 6, 1–10. 10.1186/1744-9081-6-9 20205867 PMC2826281

[B45] Stovner L. J. Al Jumah M. Birbeck G. L. Gururaj G. Jensen R. Katsarava Z.. (2014). The methodology of population surveys of headache prevalence, burden and cost: Principles and recommendations from the Global Campaign against Headache. J. Headache Pain. 15, 1–30. 10.1186/1129-2377-15-5 24467862 PMC3907133

[B46] Stovner L. J. Hagen K. Jensen R. Katsarava Z. Lipton R. B. Scher A. I.. (2007). The global burden of headache: a documentation of headache prevalence and disability worldwide. Cephalgia 27, 193–210. 10.1111/j.1468-2982.2007.01288.x 17381554

[B47] Su M. Yu S. (2018). Chronic migraine: a process of dysmodulation and sensitization. Molec. Pain. 14, 1–10. 10.1177/1744806918767697 29642749 PMC5900816

[B48] Tessitore A. Russo A. Giordano A. Conte F. Corbo D. De Stefano M.. (2013). Disrupted default mode network connectivity in migraine without aura. J. Headache Pain. 14, 1–7. 10.1186/1129-2377-14-89 24207164 PMC3832236

[B49] Uddin L. (2015). Salience processing and insular cortical function and dysfunction. Nature Reviews Neuroscience 16, 55–61. 10.1038/nrn3857 25406711

[B50] Wager T. Davidson M. Hughes B. Lindquist M. Ochsner K. (2008). Prefrontal-subcortical pathways mediating successful emotion regulation. Neuron 59, 1037–1050. 10.1016/j.neuron.2008.09.006 18817740 PMC2742320

[B51] Walker J. (2011). QEEG-guided neurofeedback for recurrent migraine headaches. Clin. EEG Neurosci. 42, 59–61. 10.1177/155005941104200112 21309444

[B52] Weiller C. May A. Limmroth V. (1995). Brain stem activation in spontaneous human migraine attacks. Nat. Med. 1, 658–60. 10.1038/nm0795-658 7585147

[B53] Womac A. Burkeen J. Neuendorff N. Earnest D. Zoran M. (2009). Circadian rhythms of extracellular ATP accumulation in SCN cells and cultured astrocytes. Eur. J. Neurosci. 30, 869–876. 10.1111/j.1460-9568.2009.06874.x 19712092 PMC2757148

[B54] Xue T. Yuan K. Cheng P. Zhao L. Zhao L. Yu D.. (2013). Alterations of regional spontaneous neuronal activity and corresponding brain circuit changes during resting state in migraine without aura. NMR Biomed. 26, 1051–1058. 10.1002/nbm.2917 23348909

[B55] Zhang J. Su J. Wang M. Zhao Y. Yao Q. Zhang Q.. (2016). Increased default mode network connectivity and increased regional homogeneity in migraineurs without aura. J. Headache Pain. 17, 1–9. 10.1186/s10194-016-0692-z 27771875 PMC5075323

